# Technological advances and clinical application of left ventricular assist devices in the treatment of advanced heart failure

**DOI:** 10.1186/s13019-026-04599-4

**Published:** 2026-07-20

**Authors:** Chenyu Liu, Yuxiang Zhu

**Affiliations:** Department of Cardiac Surgery, Weifang People’s Hospital, Shandong Second Medical University, 151 Guangwen Street, Kuiwen District, Weifang, 261000 Shandong China

## Abstract

Left ventricular assist devices (LVADs) have become an important treatment for advanced heart failure, especially given the shortage of heart transplant donors. This article systematically reviews the technological evolution, perioperative assessment, pathophysiological changes, gender differences, functional recovery, and complication management of LVADs, focusing on the CH-VAD, a fully magnetically levitated centrifugal pump independently developed in China. Results show that LVAD technology has evolved from pulsatile flow devices to the third-generation magnetically levitated centrifugal pump, significantly improving blood compatibility and treatment safety. CH-VAD has demonstrated good efficacy and safety in clinical applications, with a 6-month and 1-year survival rate of 91.6%, a low rate of drive-line infection (11.0%), and unique advantages in complex anatomical cases. LVADs can effectively improve patients’ cardiac function, quality of life, and exercise tolerance, promoting cardiac remodeling and skeletal muscle recovery, but hemodynamic regulation during exercise remains limited. The risk of complications (stroke, infection) remains higher in female patients than in male patients. The prediction and management of right heart failure remains a key challenge affecting prognosis. In the future, it is necessary to combine biomarker-guided patient screening, gender-specific risk management, and the technological iteration of domestically produced equipment to promote the individualization and precision of LVAD treatment.

## Introduction

Heart failure is a disease caused by impaired cardiac pumping function, which leads to the heart’s inability to meet the body’s basic metabolic needs. It has become a major global health problem. Epidemiological data shows that about 1.3% of people over 35 years old in China suffer from heart failure, of which about 10%-15% progress to late heart failure [1]. Although guideline-guided drug treatments (such as beta-blockers, RAAS inhibitors, aldosterone receptor antagonists, and SGLT2 inhibitors) can improve survival, many patients still develop late heart failure. Although heart transplantation is the gold standard treatment for end-stage heart failure, its application is limited due to the long-term shortage of donor organs. This gap has led to the increasing use of mechanical circulatory support, making LVAD implantation one of the core means of treating late heart failure. It can be used as a transitional bridge to heart transplantation or as an endpoint treatment for patients who are not suitable for transplantation [2]. In recent years, LVAD technology has undergone three generations of innovation, from pulsatile flow to continuous flow and then to magnetic levitation. In particular, the CH-VAD (also known as BrioVAD), a fully magnetically levitated centrifugal pump independently developed in China, was approved for marketing by the National Medical Products Administration in 2021, providing a new treatment option for patients with advanced heart failure in China. This article aims to systematically review the technical characteristics, clinical applications, perioperative assessment, pathophysiological changes, gender differences, functional outcomes and complication management of CH-VAD in the treatment of advanced heart failure, taking CH-VAD as the core and combining international LVAD technology advancements, in order to provide a reference for clinical practice and future research.

## Technological evolution of left ventricular assist devices: from pulsating flow to magnetic levitation

### Iteration of the first three generations of LVAD technology

**Fig. 1:**
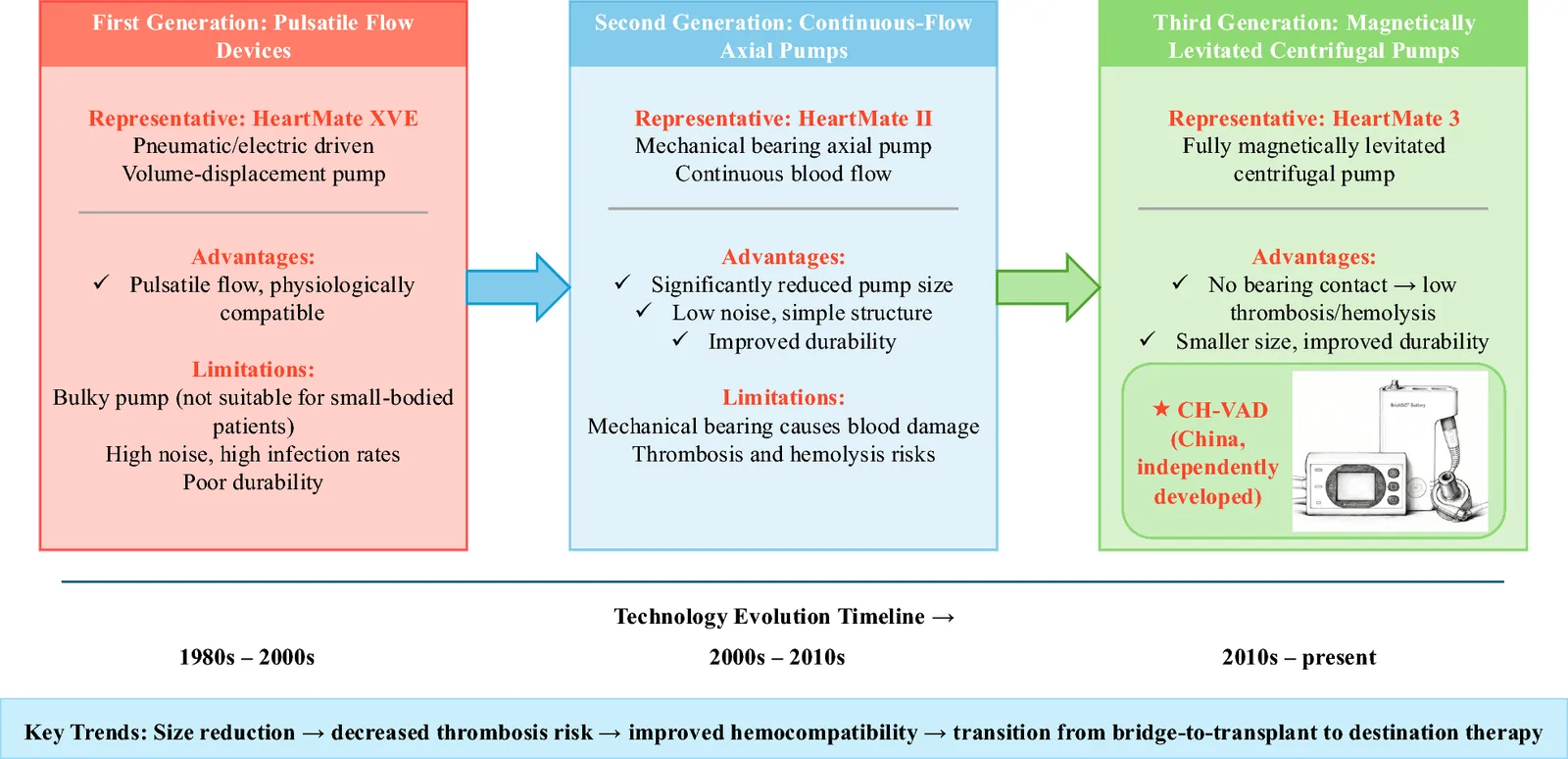
Evolution of LVAD technology and the positioning of CH-VAD

The development of LVAD has undergone three generations of technological innovation. The first generation of pulsatile flow devices (represented by HeartMate XVE) provides circulatory support by simulating the pulsatile ejection of blood from a natural heart and has good antithrombotic performance, but the pump body is large and not suitable for small patients. The second generation of continuous flow devices (represented by HeartMate II) adopts axial flow pump technology, and the pump body is significantly reduced, making it easy to implant in the abdominal wall or thoracic cavity, but the mechanical bearings still pose a risk of blood damage and thrombosis. The third generation of magnetic levitation centrifugal pumps represents the most advanced technology direction at present. Taking HeartMate 3 (HM3) as the international benchmark, this device adopts fully magnetic levitation technology (bearingless motor), and the rotor rotates in a non-contact state, which significantly reduces the risk of hemolysis and thrombosis, reduces bleeding and stroke events, and improves treatment safety .The 5-year follow-up results of the MOMENTUM 3 pivotal trial confirmed that HM3 is superior to the second generation device in terms of survival rate, the composite endpoint of avoiding stroke or reoperation, and established the status of fully magnetically levitation LVAD as the standard treatment for advanced heart failure [3]. In addition, studies have shown that HM3 implantation can immediately and continuously improve brain autoregulation function and enhance cerebrovascular reactivity at 90 days, suggesting long-term cerebrovascular adaptation [3] (Fig. 1).

### Independent innovation in China: CH-VAD fully magnetically levitated centrifugal pump

CH-VAD (developed by BrioHealth Solutions, also known as CH-VAD in China) is a new generation of fully magnetically levitated centrifugal LVAD independently developed in China. Unlike the bearingless motor of HeartMate 3, CH-VAD adopts a split motor and magnetic bearing technology, which optimizes the internal space layout of the pump and increases the internal impeller diameter while reducing the overall size of the pump body [1].

Technical features and design advantages: CH-VAD has two flow channels, a main channel and a secondary channel. The rotor drives the impeller mounted on the flat top to generate centrifugal flow. The nose cone structure can convert the axial flow into radial flow, improve the flow velocity distribution in the impeller inlet and downstream area, and make it more uniform. After the magnetic levitation is started, a stable U-shaped suspension gap (0.25 mm wide) is formed between the rotor and the annular channel of the pump casing, which serves as a secondary flow channel to balance blood flow and reduce high shear stress and turbulence exposure [1, 4].

Surgical experience: Clinical implantation feedback, CH-VAD’s compact pump design makes it easy to implant in small patients. Its innovative suture ring and outflow tube anastomosis technology simplifies the surgical procedure and helps to shorten the cardiopulmonary bypass time. This device is especially suitable for cases with complex anatomy, such as high-risk patients with aortic aneurysm, multi-vessel coronary artery disease and valvular regurgitation, where traditional treatment methods are limited, providing a feasible mechanical circulation support solution for this population [4, 5].

Clinical efficacy and safety: A large single-center study involving patients with end-stage heart failure confirmed that CH-VAD can provide effective circulatory support, significantly improve patient survival and effectively manage complications [1]. Multicenter studies showed that the 6-month and 1-year survival rates of patients reached 91.6%, and all patients’ cardiac function improved to NYHA class I/II after 6 months of support [6]. In terms of thrombotic risk assessment, numerical models suggest the existence of low flow velocity and stagnation areas in its flow channel, but compared with other devices, CH-VAD has the lowest thrombotic risk, reflecting the antithrombotic advantage of the overall design [4].

Infection control: The overall incidence of drive-line-related infection in CH-VAD is 11.0%, with Staphylococcus aureus as the main pathogen, of which 35% are deep infections [7]. By using ultra-thin flexible drive lines (only 3.3 mm in diameter) in conjunction with standardized nursing procedures, CH-VAD significantly reduces the risk of drive-line infection and helps improve the safety of supportive care [7].

Comparison with HeartMate 3: There are currently no head-to-head randomized controlled trials of CH-VAD and HM3. Based on the existing data, CH-VAD’s 6-month survival rate (91.6%) is comparable to the recent results of HM3 (the 6-month survival rate of HM3 in the MOMENTUM 3 trial was about 92%) [6]. CH-VAD has potential advantages in pump size and drive wire diameter, while HM3 has longer follow-up data and more extensive international application experience. The BrioVAD clinical trial of CH-VAD (ClinicalTrials.gov identifier: NCT06310031) is underway in the United States, which will further verify its safety and efficacy in the international population.

### Perioperative assessment and management of LVAD

#### Patient selection and preoperative assessment

Accurate patient screening is the cornerstone of successful LVAD implantation. Analyzing the characteristics of patients undergoing LVAD surgery can provide effective guidance for preoperative assessment.

Gender and age distribution: Gunawan’s (2024) study included only 32 patients using the Medtronic HeartWare LVAD, a relatively small sample size [8]. The study by Hayashi (2024) covered 188 patients who received HeartMate 3 implants, with a longer time span, showing the advantages of large sample multicenter studies [9]. In addition, Shad’s (2021) study focused on 29 patients and explored the impact of new onset aortic regurgitation on right ventricular function [10]. Multiple studies have consistently shown that LVAD recipients are predominantly male. Bravo's (2021) large cohort data showed that males accounted for 81.8% and females for only 18.2%, a distribution consistent with the gender epidemiology of heart failure [11]. In terms of age, LVAD is mainly used for middle-aged and elderly patients aged 55-60 years [12, 13]. For elderly patients aged ≥75 years, preoperative assessment of renal function (eGFR ≥45 ml/min/1.73m^2^ is the relative safety threshold), cognitive function, and comorbidities (such as COPD and diabetes) is required. Magnetic levitation devices with low thrombotic risk should be prioritized, and perioperative infection risk management should be strengthened [14, 15]. For younger patients <55 years, if it is a pre-transplant transitional therapy, a more durable device can be selected; if it is an endpoint therapy, the feasibility of complication control should be carefully assessed.

Preoperative left ventricular size and prognosis: Preoperative left ventricular size is closely related to patient prognosis. A study by Molina et al. (2025) on 1921 patients with HM3 implantation found that smaller left ventricular end-diastolic diameter (LVEDD <55 mm) was often associated with lower postoperative survival. This group of patients had unique preoperative characteristics: older age, higher proportion of females, higher proportion of ischemic causes, and worse hemodynamic parameters (greater right ventricular functional load) [16]. Therefore, preoperative measurement of LVEDD is of great significance for risk assessment.

Impact on preoperative cardiac function: Several studies have attempted to assess the impact of LVAD implantation on preoperative cardiac function, mainly related to left ventricular ejection fraction (LVEF) and functional volume. Severe ventricular dysfunction is a common preoperative pathological basis for LVAD patients, especially in heart failure patients with LVEF < 25% [17]. At the same time, their functional volume also shows a progressive decline, and a sharp drop in peak oxygen consumption (pVO₂) is an objective marker of this damage [14]. In clinical decision-making, for patients with LVEF of 15%-25% and pVO₂ of 8-12 mL/kg/min, the necessity of LVAD implantation can be directly assessed [15]. For critically ill patients with LVEF < 15% and pVO₂ < 8 mL/kg/min, short-term mechanical circulatory support (e.g., intra-aortic balloon counterpulsation) is required to optimize hemodynamics, and LVAD implantation should be performed after organ function stabilizes [18].

### Postoperative hemodynamics and blood pressure management

Blood pressure management after LVAD surgery is challenging because the continuous flow device generates non-pulsatile blood flow, making conventional blood pressure measurement and regulation more difficult. Studies have shown that the loss of cardiac pulsatility after continuous flow LVAD (CF-LVAD) implantation is a potential factor in the development of hypertension [19].

Regulation plan: After surgery, blood volume is first adjusted by intravenous diuretics; if blood pressure is still high (MAP > 95 mmHg), β-blockers can be added, and strong vasodilators should be avoided [20]. Sailer et al. (2021) monitored the blood pressure of 12 patients with heart failure with reduced ejection fraction (HFrEF) before and after CF-LVAD implantation. The results showed that the resting cardiac output returned to normal, but the blood pressure reserve under exercise was still insufficient, suggesting that the effect of CF-LVAD on blood pressure regulation differs between resting and exercise states [19] (Table 1).

**Table 1:** Comparison of hemodynamic parameters before and after implantation

Index	State	Pre-LVAD	Post-LVAD	Variation trend
Systolic blood pressure (SBP,mmHg)	rest	117 ± 11	108 ± 23	decrease
Diastolic blood pressure (DBP,mmHg)	rest	66 ± 22	78 ± 10	increase
Pulse pressure (PP,mmHg)	rest	51 ± 28	30 ± 21	significant decrease
Mean arterial pressure (MAP,mmHg)	rest	78 ± 10	88 ± 12	significant increase
Peak workload (Watt)	exercise	20 ± 21	31 ± 18	increase
Maximum oxygen intake (VO2Max)	exercise	—	—	—
Mean arterial pressure (MAP,mmHg)	exercise	79 ± 12–92 ± 16	—	increase
Pulse pressure (PP,mmHg)	exercise	—	28 ± 14–59 ± 26	significant increase

### Postoperative cardiac function, exercise tolerance, and cardiac remodeling

#### Changes in cardiac function and exercise tolerance

LVAD implantation can significantly improve the cardiac function classification of patients, and the New York Heart Association (NYHA) classification is usually improved from preoperative class III-IV to class I-II. Postoperative 6-minute walking distance (6MWD) significantly increased. A study involving 200 patients with advanced heart failure and reduced ejection fraction confirmed that LVAD increased 6MWD from 187 meters at baseline to 263 meters, an average increase of 75 meters (P < 0.001) [21, 22]. Clinical rehabilitation training can begin one month postoperatively (gradually transitioning from bedside activities to walking on flat ground) [23], with a goal of achieving a 6MWD of more than 250 meters within 6 months [24]. If 6MWD does not improve, pump function (e.g., whether the flow rate is sufficient) and complications (e.g., anemia, infection) should be evaluated [25].

However, despite improvements in quality of life, the recovery of objective cardiopulmonary function indicators remains limited. A study by Buchanan et al. (2024) found that there was no statistically significant difference in pVO₂ after LVAD implantation compared with preoperative values (P = 0.92), far from reaching the health standard of >20 mL/kg/min. Under exercise load, the patients’ hemodynamic parameters remained abnormal: pulmonary capillary wedge pressure (PCWP) increased from 10 mmHg at rest to 20 mmHg at peak exercise (P < 0.01), and the postoperative (fixed/dynamic pump rate) peak exercise PCWP still reached 25/21 mmHg, respectively; peak exercise power increased from 19 W to 34 W (P = 0.24), but pVO₂ did not increase synchronously, indicating that peripheral tissue oxygen utilization was not improved. This shows that although LVAD improved the patients’ daily activity tolerance, it failed to correct the abnormal cardiac chamber filling pressure and impaired maximum oxygen uptake during exercise, highlighting the limitations of the current device in optimizing hemodynamics under exercise [14, 26]. Postoperative patients’ pVO₂ usually only recovered to about 50% of the normal level, indicating that although cardiac function has improved, it has not fully recovered to normal [14, 27].

### Myocardial remodeling

LVAD implantation reduces ventricular wall stress through continuous ventricular unloading, reversing the vicious cycle of heart failure. Structural remodeling is mainly manifested as: a significant reduction in left ventricular end-diastolic diameter (optimal target <60 mm), a reduction in myocardial mass, and an improvement in the degree of mitral regurgitation (≤ moderate). The hemodynamic stress test (ramp test) sets clear criteria for ventricular unloading: maintaining PCWP below 18 mmHg, central venous pressure below 12 mmHg, and cardiac index above 2.2 L/min/m^2^ [28–30].

Histological and molecular level remodeling: LVAD support can induce a reduction in cardiomyocyte hypertrophy, a reduction in myocardial fibrosis, and extracellular matrix remodeling. Postoperative dynamic monitoring of significant decreases in heart failure biomarkers (such as NT-proBNP, ST2, GDF-15) is an important biological indicator of reduced myocardial load, improved inflammation and fibrosis [30]. These changes provide a potential biological basis for postoperative weaning or recovery.

### Skeletal muscle mass recovery

**Fig. 2:**
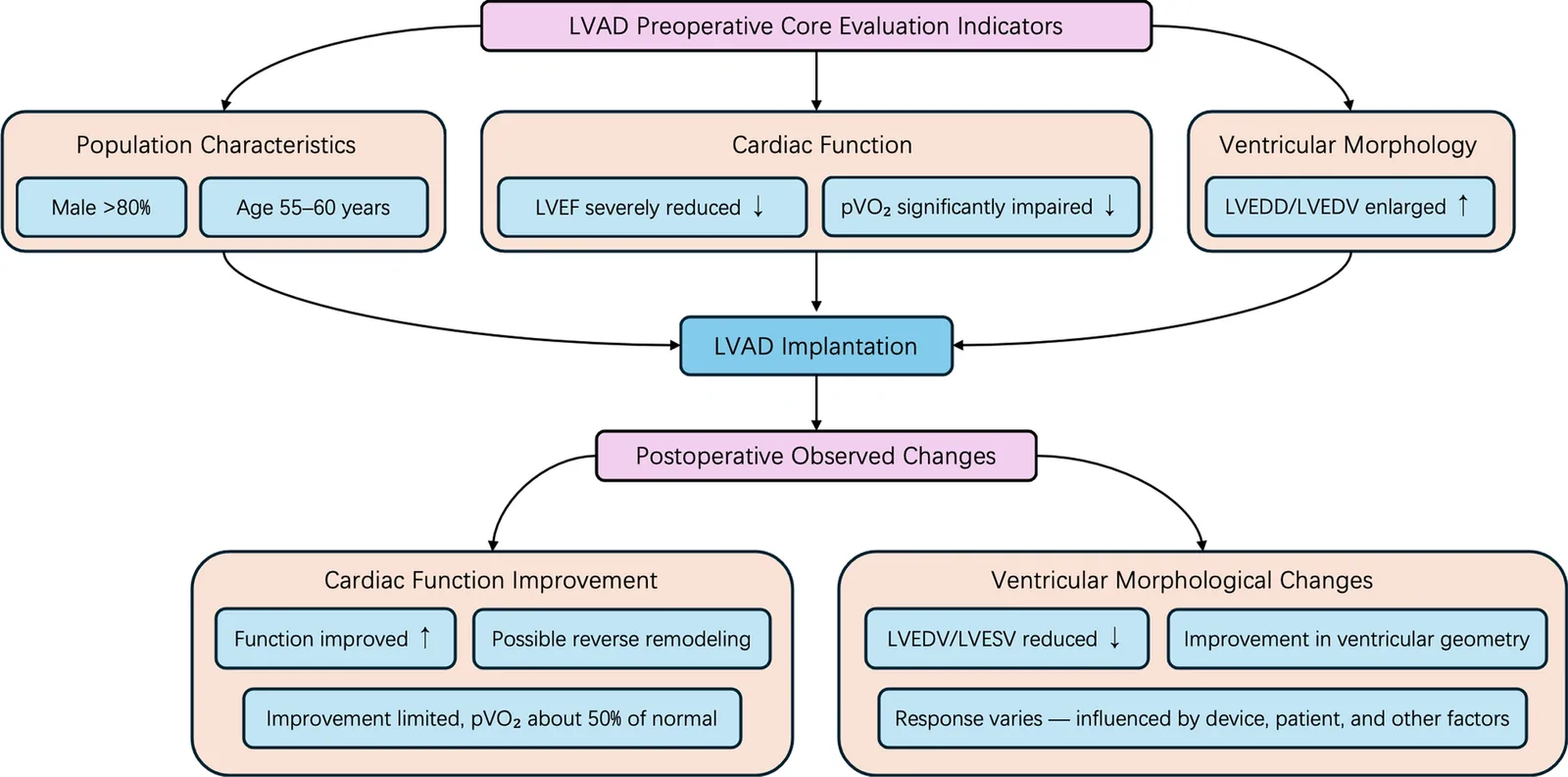
LVAD preoperative core evaluation indicators

Patients with advanced heart failure often have skeletal muscle atrophy, but studies have shown that this atrophy is reversible after LVAD support. Observational data within six months after surgery showed that, measured by dual-energy X-ray absorptiometry, patients showed significant increases in fat-free body mass and skeletal muscle mass at 3 and 6 months of supportive treatment, demonstrating that muscle atrophy can be improved 3-6 months after intervention [31] (Fig. 2) (Table 2).

**Table 2:** General condition of the patient

Research authors (year)	Sample size (n) and equipment type	Average age (years) and gender ratio	Underlying diseases, grouping, and preoperative characteristics	Key findings and outcomes
Gunawan (2024)	32 cases Medtronic HeartWare (HVAD)	56.0 ± 12.8 Male: 81.3%	History of heart failure: > 2 years (75%) Etiology: Non-ischemic (65.6%) Implantation purpose: All for bridging to heart transplantation (BTT)	**Postoperative improvement:** At 3 and 6 months follow-up, BMI, creatinine, and hemoglobin significantly increased, while the proportion of diuretic use decreased (88.5% to 68.2%)
Hayashi (2024)	188 cases Equipment type unspecified	59 ± 12 Male: 84%	**Grouping:** Based on the changes in right ventricular end-diastolic diameter/area (RVEDD/RVEDA), the groups were divided into: Non-adaptive right ventricle group (NARV, n = 82, 44%) Adaptive right ventricle group (n = 106, 56%) **Preoperative characteristics:** The NARV group had a lower pulmonary capillary wedge pressure (PCWP)	The NARV group more commonly underwent median sternotomy There were no differences in cardiac index and pulmonary artery pulsatility index between the two groups Postoperative echocardiography showed that the NARV group had greater right heart dimensions compared to the adaptive group
Shad (2021)	29 cases HeartMate II, HeartWare	AI group:62.2 ± 9.7 Non-AI group:55.4 ± 14.0 Male: 74%	**Grouping:** Based on the presence or absence of postoperative aortic valve insufficiency (AI), the groups were divided into: AI group (n = 9) Non-AI group (n = 19) **Preoperative characteristics:** There were no significant differences between the two groups in age, sex, BMI, mean arterial pressure, or device type selection	There were no statistical differences in baseline characteristics between the AI group and the non-AI group preoperatively
Bravo (2021)	525 cases No difference in device type	Median age: 60.6 (49.6–68.1) Predominantly male	**Grouping:** Based on the presence or absence of intracardiac thrombus (ICT), the groups were divided into: ICT4E group (n = 44, 8.4%) No ICT group (n = 481) **Preoperative characteristics:** The ICT group was younger, with a higher history of stroke, higher INR, higher white blood cell count, lower albumin levels, and a greater number of patients classified as INTERMACS level 1	The stroke rate (18.2% vs 7.3%) and 6-month mortality rate (22.7% vs 9.4%) were significantly higher in the ICT group Stroke is one of the main causes of death
Buchanan (2024)	10 cases Equipment type unspecified	60 ± 10 Predominantly male	**Population:** Patients with advanced heart failure with reduced ejection fraction (HFrEF) **Preoperative characteristics:** Average BMI 31 ± 8 kg/m^2^, average body surface area (BSA) 2.1 ± 0.4 m^2^	Detailed anthropometric data showed that weight, BMI, and BSA all increased after LVAD implantation
Molina (2025)	1921 cases HeartMate 3	LVEDD < 55 mm group:63 ± 11 LVEDD ≥ 55 mm group:60 ± 12 Male: < 55 mm group: 69%, ≥ 55 mm group: 80.2%	**Grouping:** Based on left ventricular end-diastolic diameter (LVEDD), the groups were divided into: LVEDD < 55 mm group (n = 108) LVEDD ≥ 55 mm group (n = 1813) **Preoperative characteristics:** The < 55 mm group had more females, smaller BSA, higher proportion of ischemic etiology, lower CRT/ICD implantation rate, higher LVEF, lower PAPi, and higher RAP/PCWP	The unique preoperative characteristics of patients with a "small left ventricle" (LVEDD < 55 mm) were defined: older age, more females, higher proportion of ischemic etiology, and poorer hemodynamic parameters (greater right heart functional load)

### Clinical outcomes and complication management of LVAD

#### Gender differences in clinical outcomes

Survival Outcomes: In the MOMENTUM 3 study, Ramu et al. (2023) reported that 2 years after HeartMate 3 implantation, there were no significant differences between men and women in key clinical outcomes, including the composite endpoint of no disabling stroke or reoperation and overall survival [32]. Steinberg et al. (2024) analyzed the OPTN database and found that when HM3 was used as a pre-transplant bridging therapy, gender did not affect the survival rate of patients waiting for transplantation or 1 year after transplantation [33]. This suggests that the new generation of LVADs may have narrowed the overall survival differences between men and women, and gender should not be used as a clinical contraindication for LVAD implantation.

Complications and Adverse Events: Despite similar overall survival rates, Ramu et al. (2023) pointed out that female patients (especially younger patients) had a significantly higher risk of stroke and serious infection than men [32]. The national database study by Shetty et al. (2023) also found that women had a higher overall incidence of adverse events after LVAD treatment, and this phenomenon was consistent across different clinical subgroups [34]. These results indicate that gender differences associated with blood compatibility and infection risk remain key challenges in the management of female patients.

**Fig. 3:**
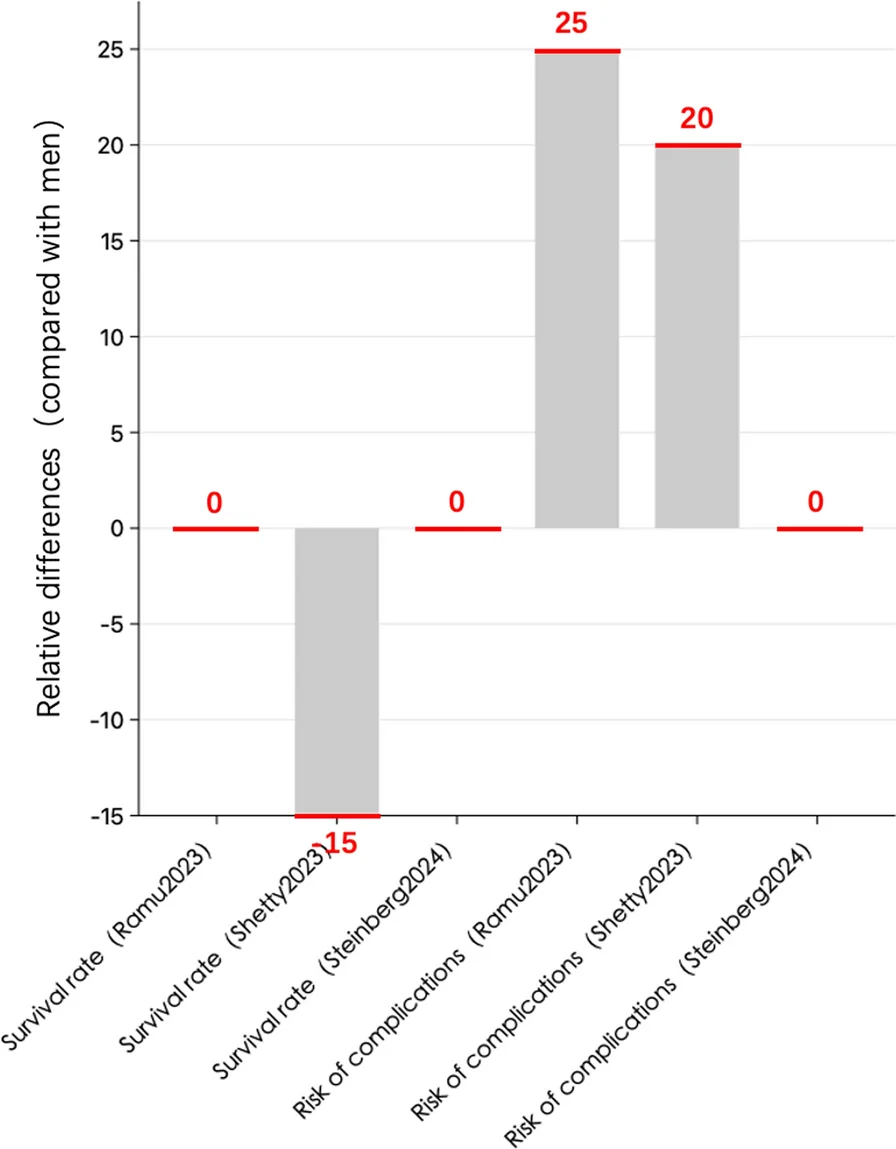
Gender differences in clinical outcomes of LVAD: comparison of three studies

The results of these three studies corroborate each other. These findings indicate that with the application of new-generation LVADs, the overall survival gap between men and women is narrowing, but women still face a higher risk of complications. Therefore, future research needs to pay more attention to gender differences and strive to develop risk management strategies specifically for women (Fig. 3).

### Prediction and management of right heart failure

Right heart failure (RHF) is one of the most common and prognostic complications after LVAD, and it occurs in the early stage (within 14 days after surgery) and the late stage (after 14 days after surgery).

The pathogenesis of late right heart failure can be summarized as preoperative intrinsic factors and postoperative related factors. Among the preoperative factors, primary cardiomyopathy involving the right ventricle (such as ischemic or dilated cardiomyopathy) is an important cause of late RHF. Among the postoperative factors, some patients still have moderate to severe mitral regurgitation after LVAD implantation, which is closely related to the increased risk of right ventricular dysfunction, right ventricular enlargement, and systolic dysfunction [35]. The inability to effectively control regurgitation through pump rate may accelerate the progression of pulmonary vascular disease, eventually leading to late RHF. In addition, changes in interventricular septum morphology caused by postoperative left ventricular unloading are also an important mechanism leading to late RHF [36–38].

Predictive value of right ventricular myocardial work: Studies have found that low overall right ventricular work efficiency is an important factor leading to early RHF and can serve as an early warning signal of the disease. Adding right ventricular myocardial function assessment (such as right ventricular myocardial work index RVMW) to the preoperative screening of LVAD indications can improve the accuracy of patient screening and play a positive role in preventing early postoperative RHF [39].

Clinical recommendations: In the routine preoperative screening process, it is recommended to include right ventricular myocardial work (RVMW) and right ventricular-pulmonary artery coupling parameters (such as fwRVLS/sPAP) in the assessment system [40, 41].

### Other major complications

Driver line infection: This is the most common type of infection after LVAD. The incidence of driver line-related infection in the CH-VAD cohort was 11.0%, with Staphylococcus aureus being the main pathogen [7]. Prevention strategies include: ultra-thin flexible drive line design, standardized exit care procedures, and avoidance of exit site trauma.

Aortic regurgitation: Long-term aortic valve insufficiency caused by continuous unloading of the LVAD can induce or aggravate aortic regurgitation (AI) [42, 43]. Shad et al. (2021) found through patient-specific computational fluid dynamics (CFD) studies that local blood flow patterns can predict postoperative new AI, providing new ideas for preoperative risk assessment and intraoperative valve management [10].

Gastrointestinal bleeding: The mechanism of occurrence is related to the imbalance of vasodilators (such as angiotensin II and vascular endothelial growth factor), which is especially common in patients with continuous flow LVAD. Clinical management includes reducing the pump rate to increase pulsatility and using drugs such as octreotide [44, 45].

Thrombosis, hemolysis, and stroke: The full magnetic levitation design of HM3 reduces the incidence of pump thrombosis to an extremely low level (<1% in MOMENTUM 3). The numerical model of CH-VAD also shows that its thrombosis risk is lower than that of other devices [4]. Stroke events are more common in female patients, and anticoagulation management and blood pressure control need to be strengthened.

### Future prospects

#### Personalized treatment based on cardiac biomarkers

Preoperative screening (identifying potentially recoverable patients): Low levels of inflammatory biomarkers (such as interferon-γ and tumor necrosis factor-α) before surgery are closely related to cardiac remodeling and functional improvement after LVAD implantation, and can serve as an important basis for screening patients with high myocardial recovery potential and those who may be weaned off the ventilator in the future [30].

Postoperative monitoring and recovery assessment (confirming recovery trends): For patients identified as potentially recoverable, dynamic changes in heart failure biomarkers, including NT-proBNP, GDF-15, and ST2, should be closely monitored after LVAD implantation [30]. Significant reversal (decline) of these biomarkers under LVAD support is an important biological indicator of myocardial load reduction, inflammation, and fibrosis improvement.

### Technological improvement and performance optimization

Improve hemodynamic regulation during exercise: Current LVADs still have limitations in terms of exercise tolerance, cardiac filling pressure, and hemodynamic regulation. In the future, the device design should be further optimized to enhance its ability to dynamically regulate blood pressure, cardiac output, and cardiac load during exercise.

Reducing the Risk of Complications: Female patients still face a higher risk of stroke, infection, etc. [32]. Future research on gender differences is needed to develop targeted prevention and control strategies. At the same time, the biocompatibility of the device should be continuously improved to reduce the incidence of thrombosis, hemolysis, and infection.

### Development path of LVAD in China: challenges and opportunities of localization

The successful development of CH-VAD marks China’s independent breakthrough in the core technology of LVAD (magnetic levitation pump design), and the safety of the device has been gradually verified through localized clinical practice (management of high-risk patients, optimization of anticoagulation regimens) [1]. Currently, several domestically developed LVADs in China have entered the clinical application stage, among which CH-VAD was approved for marketing by the NMPA in 2021. At the regulatory level, China follows a gradual regulatory path of "consent to use - clinical trials - marketing approval", which ensures both the efficiency of innovation transformation and adherence to the bottom line of safety. With the increase of domestically produced equipment, the regulatory system is expected to be further improved [1].

Current Challenges: Clinical adoption is still limited by patient characteristics (mainly patients with dilated cardiomyopathy aged 50-57 years, with a lower proportion of ischemic causes than in Europe and the United States), distribution of medical resources (implantation centers are concentrated in large cardiac centers in first-tier cities), and factors such as patient education and acceptance [1].

**Fig. 4:**
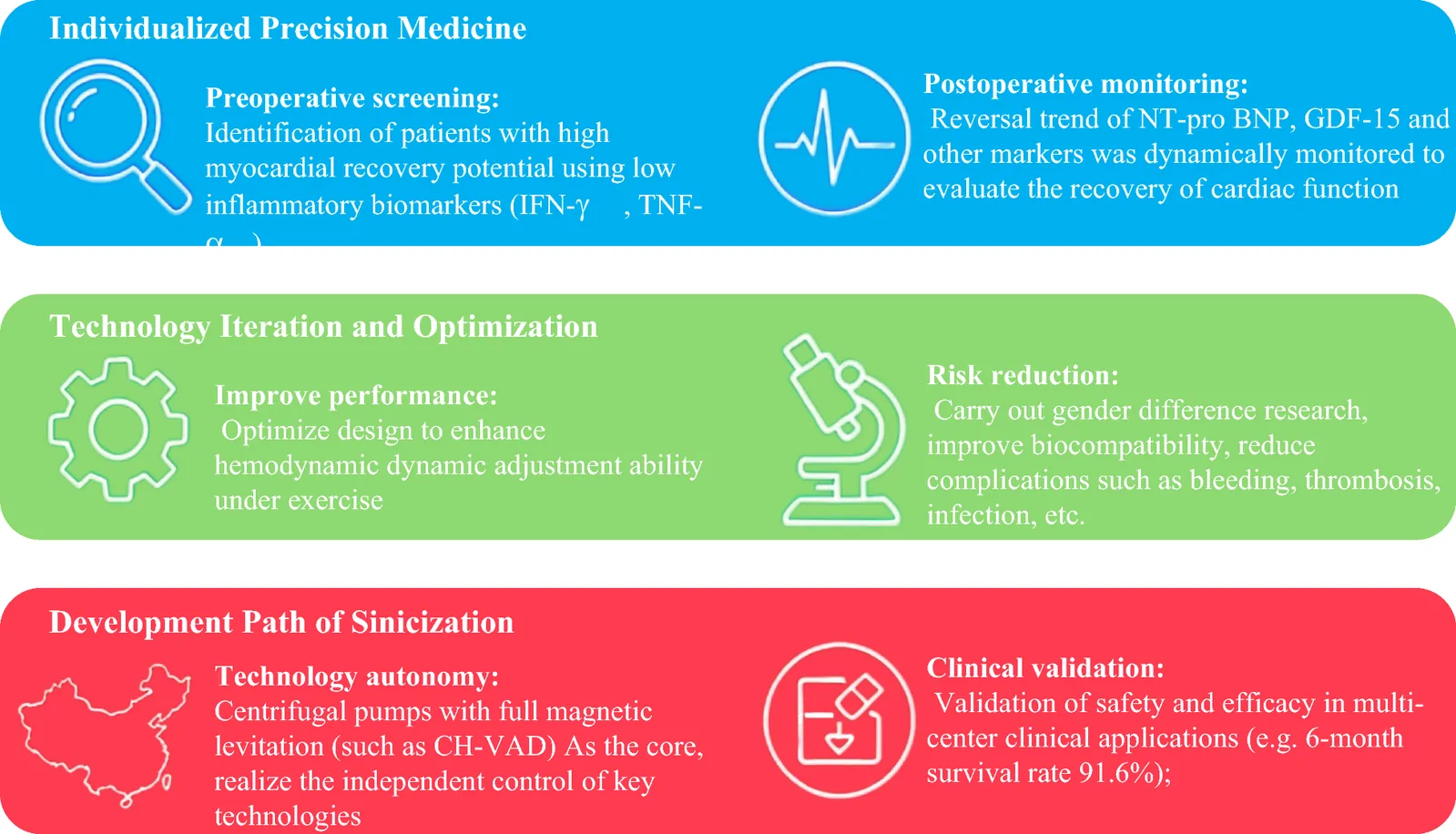
Future perspective of LVAD

Future actions: It is necessary to improve the efficacy data of elderly and complex cases, realize the independent control of the technology throughout the entire cycle, and make LVAD an important part of China’s end-stage heart failure treatment system through measures such as expanding the coverage of medical resources, strengthening physician training and patient education, deepening multidisciplinary collaboration, and promoting the coverage of medical insurance policies (Fig. 4).

## Conclusion

Left ventricular assist devices have become an important means of treating advanced heart failure, especially in the context of a shortage of heart transplant donors. This article systematically reviews the key advances of LVAD in terms of technology, clinical application, perioperative assessment, pathophysiological changes, gender differences, functional outcomes, and complication management. Current LVAD technology has evolved from early pulsatile flow devices to third-generation magnetically levitated centrifugal pumps, significantly improving blood compatibility and treatment safety. The CH-VAD device independently developed in China has shown good efficacy and safety in clinical practice, providing new treatment options for heart failure patients in China. Clinical studies have confirmed that LVAD can effectively improve patients’ cardiac function, quality of life, and exercise tolerance, and promote cardiac remodeling and skeletal muscle recovery. However, LVAD still faces challenges such as limited hemodynamic regulation during exercise, a higher risk of complications in female patients, and the prediction and management of right heart failure. Future research should combine biomarker-guided patient screening, gender-specific risk management, and the technological advancement of domestically produced equipment to promote the individualization and precision of LVAD treatment, ultimately achieving safer and more effective supportive care for heart failure.

## Data Availability

No datasets were generated or analysed during the current study.
